# Decrease of insoluble glucan formation in *Streptococcus mutans* by co-cultivation with *Enterococcus faecium* T7 and glucanase addition

**DOI:** 10.1007/s10529-017-2478-z

**Published:** 2017-11-21

**Authors:** Shin-Hye Yu, So-Hyung Kwak, Thi Thanh Hanh Nguyen, Ye-Seul Seo, Chaeri Song, Il Kyoon Mok, Doman Kim

**Affiliations:** Graduate School of International Agricultural Technology and Institutes of Green Bioscience & Technology, Seoul National University, Pyeongchang-gun, Gangwon-do 25354 South Korea

**Keywords:** *Enterococcus faecium*, Glucanase, Insoluble glucan, *Streptococcus mutans*

## Abstract

**Objectives:**

To develop preventive canine oral health bio-materials consisting of probiotics and glucanase to reduce insoluble glucan and volatile sulfur compound formation.

**Results:**

Co-cultivation of *Enterococcus faecium* T7 with *Streptococcus mutans* at inoculation ratio of 3:1 (v/v) resulted in 25% reduction in the growth of *Streptococcus mutans*. Amounts of soluble and insoluble glucans produced by *S. mutans* were decreased to 70 and 55%, respectively. Insoluble glucan was decreased from 0.6 µg/ml in *S. mutans* culture to 0.03 µg/ml in *S. mutans* co-cultivated with *E. faecium* T7 in the presence of *Lipomyces starkeyi* glucanase. Volatile sulfur compound, a main component of halitosis produced by *Fusobacteria nucleatum*, was decreased by co-cultivating *F. nucleatum* with *E. faecium.*

**Conclusion:**

*E. faecium* and glucanase can be combined as potentially active ingredients of oral care products for pets by reducing plaque-forming bacteria growth and their by-products that cause cavity and periodontal disease.

**Electronic supplementary material:**

The online version of this article (10.1007/s10529-017-2478-z) contains supplementary material, which is available to authorized users.

## Introduction

Periodontal disease is a common problem occurring in companion animals because pets have insufficient dental care routine. They are exposed to diverse microbes. Thus, pet owners use many strategies to improve the oral health of their pets. Treatment for canine oral diseases is usually expensive. In addition, chemical therapy can induce side effects and drug tolerance (Gorrel et al. 2013). Among bacteria that cause periodontal disease*, Streptococcus mutans* can synthesize insoluble glucan (mutans) using sucrose. Mutans is involved in initial dental plaque formation following colonization of periodontal bacteria (Takahashi and Nyvad 2011). Dental plaque is a biofilm consisting a group of microorganisms embedded in a matrix mainly containing carbohydrates. The glucan is composed of α-(1 → 3), α-(1 → 4), and/or α-(1 → 6)-D glucosidic linkages (Takahashi and Nyvad 2011). Hydrolysis of these linkages by using enzymes has been used to remove dental plaque (Ryu et al. 2000). *Fusobacterium nucleatum* is a major producer of halitosis (Krespi et al. 2006) due to production of volatile sulfide compounds (VSCs) such as H_2_S, methyl mercaptan (CH_3_SH), and dimethyl sulfide [(CH_3_)_2_S] by bacterial metabolism (Krespi et al. 2006).

*Enterococcus faecium* belongs to a group of lactic acid bacteria (Franz et al. 2003). It can be isolated from fermented foods such as sausage, cheese, and fermented vegetables (Giraffa 2003). Inhibitory effects of *E. faecium* on biofilm formation by cariogenic streptococci have been reported (Kumada et al. 2009; Suzuki et al. 2011). Kumada et al. (2009) have reported that culture supernatant from *E. faecium* can directly inhibit *S. mutans* biofilm formation. Its inhibition activity is associated with inhibition of *E. faecium* bacterial cells on *S. mutans* strains. Suzuki et al. (2011) have shown that *E. faecium* in dual cultures possesses bacteriostatic or bactericidal activity against *S. mutans* JCM5705, *S. mutans* Xc, and *S. sorbinus*.

Our previous study has revealed that an endo-glucanase of *Lipomyces starkeyi* can inhibit formation of water-insoluble glucan or mutan (Ryu et al. 2000). However, it is currently unclear whether co-cultivation of *E. faecium* with *S. mutans* in the presence of *L. starkeyi* endo-glucanase could reduce the formation of water-insoluable glucan from *S. mutans* or the amount of VSCs produced by *F. nucleatum*. Therefore, the objective of this study was to determine the effect of co-cultivation with *E. faecium* in the presence or absence of *L. starkeyi* endo-glucanase on amounts of VSCs produced by *F. nucleatum*, growth of *S. mutans*, and insoluble glucan formation. Results of this study could provide potential preventive materials to improve canine oral health.

## Materials and methods

### Bacterial strains and culture conditions

*Streptococcus mutans* KCTC3067 was obtained from Korean Collection for Type Cultures. *F. nucleatum* KCOM 1250 was obtained from Korean Collection for Oral Microbiology (KCOM, Korea). *S. mutans* was cultured in brain heart infusion media (BHI) containing sucrose (20 g/l). *F. nucleatum* was cultured in BHI media containing yeast extract (5 g/l), hemin (5 mg/l), and vitamin K (0.2 mg/l). They were cultured at 37 °C for 2 days in 80% (v/v) N_2_, 15% (v/v) CO_2_, and 5% (v/v) H_2_ in a gas jar (Oxoid Ltd, England) with a paper sachet (Anaero Gen sachet, AN0025, OXOID Ltd, England). *Lipomyces starkeyi* glucanase (4.4 U dextranase activity/ml and 0.27 U mutanase activity/ml, respectively) was prepared as describe previously (Ryu et al. 2000).

### Isolation and identification of microorganism

*E. faecium* T7 was isolated from kimchi and incubated at 37 °C for 48 h on de Man Rogosa Sharpe (MRS) agar. To identify the strain, 16S rRNA analysis was performed using universal primers 27F (5′-AGAGTTTGATCCTGGCTCAG-3′) and 1492R (5′-GGTTACCTTGTTACGACTT-3′). Additional Biolog GEN III micro test was performed for phenotypic analysis as described previously (HarrisBaldwin and Gudmestad 1996). Development of color was observed using a micro-plate reader at 590 nm until a similarity index (SIM) was around 0.5. Species identification was made using reference metabolic profiles available in the Biolog GEN III database (version 5.2.1).

### Beaker-wire test to determine insoluble glucan formation

Beaker-wire tests were performed as described previously (Chung et al. 2004). Briefly, equal amounts of *S. mutans* and *E. faecium* T7 isolates (10^6^ CFU/ml) were co-cultured in a vial containing 10 ml test medium containing a mixture of equal volume of BHI and MRS with 20 g sucrose/l and 100 mM MOPS (Cutt et al. 2007). Three stainless steel wires (5 cm length, 1 mm diam.) were immersed in each vial and incubated at 37 °C for 24 h. Each wire was then weighed.

### Co-cultivation of *E. faecium* T7 with *S. mutans*

To determine the effect of *E. faecium* T7 co-cultivation on growth of *S. mutans*, culture medium was prepared with the same volume of MRS and BHI media containing sucrose (50 g/l, pH 6.5). Using each seed-culture after overnight growth, *S. mutans* (2.8 × 10^8^ CFU/ml) and *E. faecium* T7 (8.1 × 10^10^ CFU/ml) were mixed and inoculated at different ratios [10:0 (*S. mutans* control), 3:1, 1:1, or 1:3 (v/v)] and incubated at 37 °C for 12 h with gentle shaking (110 rpm). Then, we plated serially diluted co-culture broth on BHI agar plates containing 50 g sucrose/l and incubated at 37 °C for 24 h. *S. mutans* formed glucans by using sucrose. Therefore, the mucous *S. mutans* colonies were distinguished from *E. faecium* (Supplementary Fig. 1). Relative survival rate of *S. mutans* was obtained using the following equation:

$$ {\text{Relative}}\,{\text{Survival}}\,{\text{of}}\,S.\,mutans\,\left( \% \right) = \,\frac{{{\text{CFU}}\,{\text{of}}\,S.\,mutans\,{\text{coincubated}}\,{\text{with}}\,E.\,faecium\,{\text{T7}}}}{{{\text{CFU}}\,{\text{of}}\,S.\,mutans\,{\text{control}}}}\,\, \times \,100 $$

### Inhibitory effect of co-cultivation with *E. faecium* T7 on insoluble glucan formation by *S. mutans*

Amounts of soluble and/or insoluble glucan formation and sucrose consumption patterns by *S. mutans* were determined by TLC. After co-cultivation, cell culture was centrifuged at 12,000×*g* for 30 min. The TLC plate (silica gel 60 F_254_) was then spotted with 1 μl co-culture supernatant. Culture medium was centrifuged and the pellet was washed twice with distilled water to remove the residual media. After hydrolysis with 1 M NaOH, 1 µl suspended pellet was spotted onto a TLC plate which was then developed with two ascents of acetonitrile/water (85:15, v/v). The developed plate was dried and dipped into 0.3% (w/v) *N*-(1-naphthyl)ethylenediamine dihydrochloride and 5% (v/v) H_2_SO_4_ in methanol followed heating at 120 °C for 7 min. Concentrations of soluble and/or insoluble glucan or unreacted sucrose were determined as integrated density values using AlphaEaseFC 4.0 image program (Alpha Inotech, CA, USA) with dextran or sucrose as standard as described previously (Mukerjea et al. 1996).

### Effect of co-culture of *S. mutans* and *E. faecium* T7 with additional *Lipomyces* glucanase on insoluble glucan formation

*L. starkeyi* glucanase was prepared as described previously (Ryu et al. 2000). Its activity was assayed by incubating the enzyme with 1% (w/v) dextran at 30 °C for various times (Ryu et al. 2000). Standard glucanase assay was performed to determine dextranase activity equivalent using enzyme reaction digest containing 20 μl 1% dextran, 22 μl distilled water, and 0.25 μl glucanase. To stop the reaction, 10 μl 1 M NaOH was added. After adding 148 μl copper solution, absorption at 570 nm was measured to determine the amount of reducing sugar using 96-well plate and a spectrophotometer as described previously (Fox and Robyt 1991). *E. faecium* was cultured in 5 l MRS broth at 37 °C for 18 h. Cells were centrifuged at 6780×*g* for 15 min and washed several times with distilled water to remove the residual media. Cells were then lyophilized at − 80 °C. After 0.4 g *E. faecium* T7 (10^9^ CFU/g) and 0.2 g glucanase (22 U dextranase equivalent activity/ml) were mixed in the tube and incubated at room temperature (23 °C) for 2 weeks, cell viability (CFU/ml) was then determined. Dextranase activity was measured based on the release of reducing sugar from dextran using 3,5-dinitrosalicylic acid method (Dols et al. 1997).

### Inhibitory effect of *E. faecium* T7 co-culture on the production of volatile sulfur compounds by *F. nucleatum*

To determine the inhibitory effect of co-culture with *E. faecium* T7 on the production of VSCs by *F. nucleatum*, an equal volume of each strain at 10^9^ CFU/ml was mixed together and vortexed for 10 s followed by incubation at 37 °C with gentle shaking (at 110 rpm). Then two ml growth medium (pH 7) containing 0.1% (w/v) cysteine, 0.2% (w/v) FeSO_4_, and 100 mM MOPS was carefully added into the mixed culture followed by incubation at 37 °C for 48 h under anaerobic condition as described previously (Langendijk et al. 1999). H_2_S production was assessed by determining the degree of appearance of insoluble black iron sulfide (FeS) precipitate in the test tube.

### Statistical analysis

All data are presented as mean ± standard error of the mean (SEM) from three independent experiments. In each experiment, the test was performed in triplicates. Differences between groups were determined using one-way analysis of variance (ANOVA) followed Tukey HSD method. SPSS version 23.0 for Windows (SPSS Inc., Chicago, IL, USA) was used for all statistical analyses. Statistical significance was considered at *p* < 0.05. Significantly different insoluble glucan formation was indicated by different superscripts in lower case in Tables.

## Results and discussion

### Strain isolation, biochemical characterization, and identification

The 16S rRNA sequence from T7 strain isolated from kimchi shared 99% sequence identities with *E. faecium* 16S rRNA sequence (GenBank Accession No: CP006030.1). Based on biochemical characteristics determined with Biolog system using 73 substrate oxidation tests and 21 sensitivity tests (Supplementary Tables 1 and 2), this T7 isolate was identified as *E. faecium* (SIM index: 0.76). *E. faecium* is commensal in human intestines. It has been used as a probiotic in both animals and human (Franz et al. 2011). Probiotics are live microorganisms that support healthy GI tract. They can also improve health condition of immunity, digestion, and stool quality (Franz et al. 2011).

### Inhibitory effect of *E. faecium* T7 co-cultivation on formation of *S. mutans* insoluble glucan

**Fig. 1:**
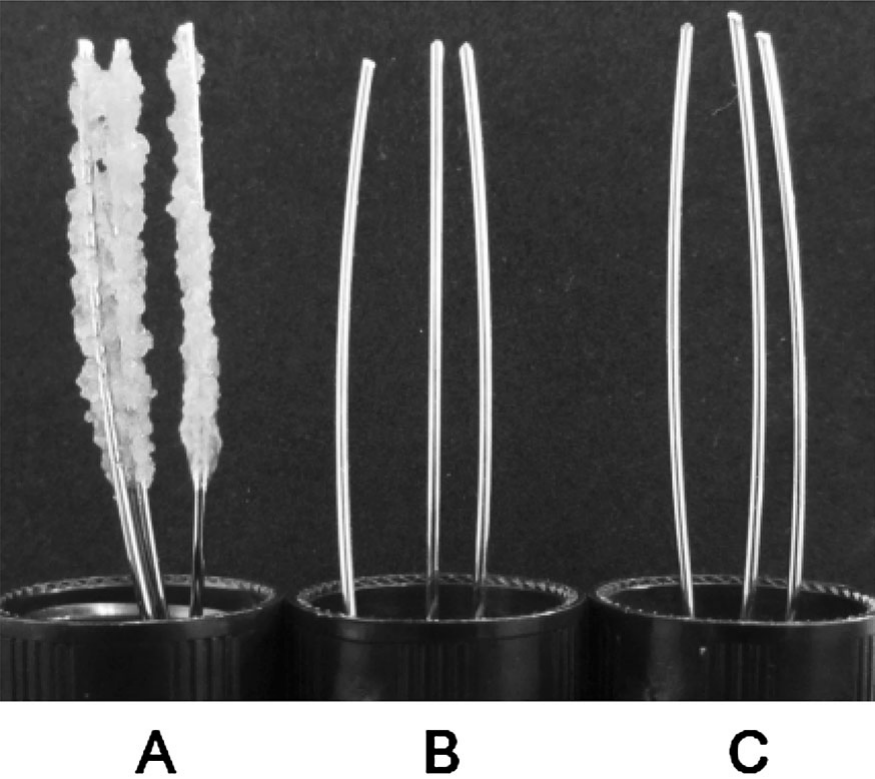
Effects of *E. faecium* T7 co-cultivation on amounts of insoluble glucans formed by *S. mutans* on wires using BHI media containing sucrose. (A) Insoluble glucans formed on wires in *S. mutans* single culture, (B) Insoluble glucans formed on wires in *E. faecium* single culture, (C) Insoluble glucans formed on wires in *S. mutans*/*E. faecium* co-culture

**Fig. 2:**
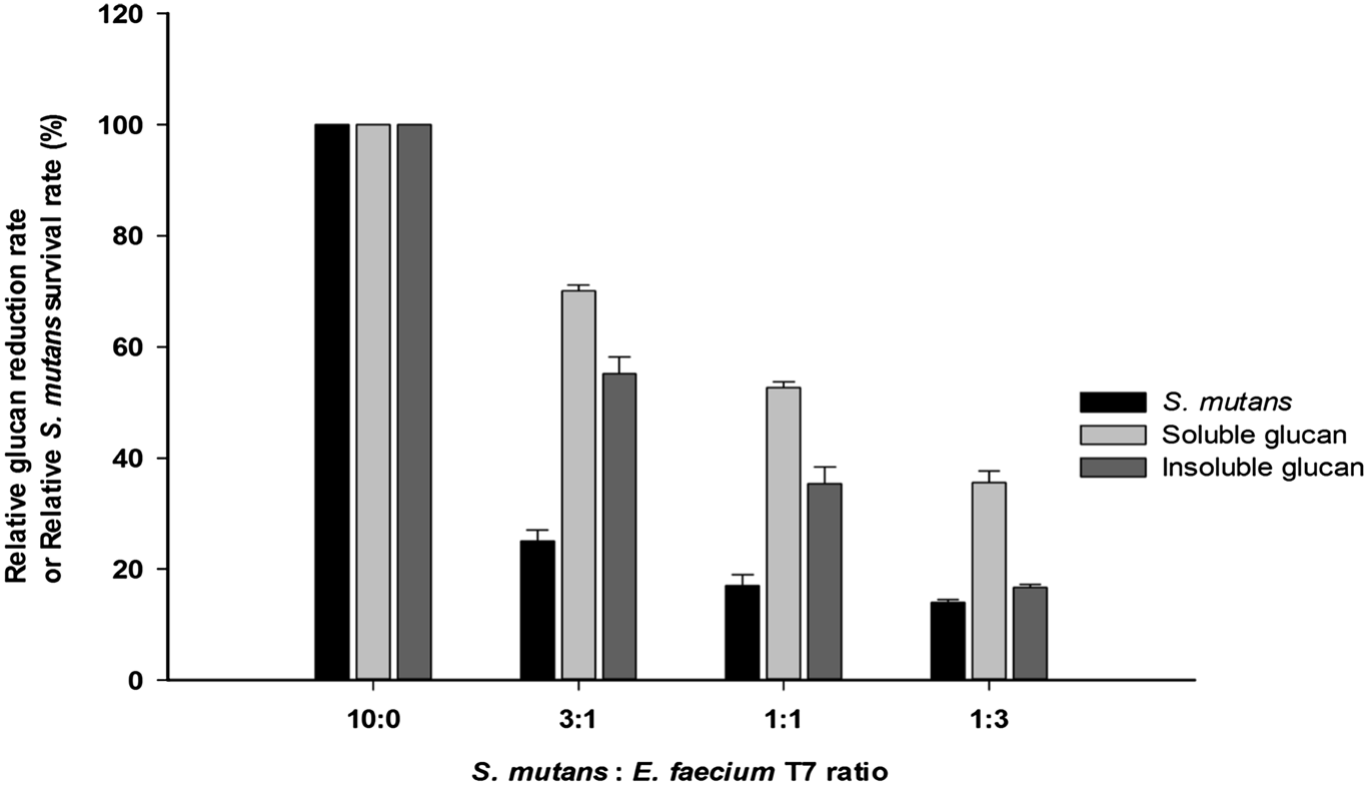
Relative growth (%) of *S. mutans* and formation of soluble and insoluble glucans in co-cultures of *S. mutans* and *E. faecium* T7 at various inoculation ratios (*S. mutans*/*E. faecium* T7 at 10:0 (v/v), 3:1 (v/v), 1:1 (v/v), and 1:3 (v/v)

Based on modified beaker and wire test, the mean weight of artificial biofilm formed on orthodontic wires was 333 mg in the group with *S. mutans* single culture. However, in the co-cultivation group, insoluble glucan was not attached onto the wire (Fig. 1). CFU after co-culture of *S. mutans/E. faecium* T7 at 3:1 (v/v), 1:1 (v/v), or 1:3 (v/v) was decreased to 25, 17, or 14% of the control (*S. mutans* single culture), respectively (Fig. 2). *E. faecium* T7 co-culture also decreased both soluble and insoluble glucans formation in sucrose medium. After co-culture, amounts of soluble glucan and insoluble glucans released to the culture media were decreased to 70 and 55%, respectively, in co-culture of *S. mutans* and *E. faecium* T7 at inoculation ratio 3:1 (v/v). They were decreased to 53 and 35%, respectively, at inoculation ratio of 1:1 (v/v) and 40 and 17%, respectively, at inoculation ratio of 1:3 (v/v) (Fig. 2).

### Effect of co-culture of *S. mutans* and *E. faecium* T7 with the addition of *Lipomyces* glucanase on insoluble glucan formation

Equal volumes of MRS and BHI broth media were mixed. The final concentration of sucrose (pH 6.5) was 5% (w/v). The final pH in each cultivation at 25 °C was 4.6. Two types of product were obtained: soluble dextran from supernatant and insoluble polymer in pellet. With increasing ratio of *E. faecium* added into the co-culutre, less insoluble and soluble glucans were formed. With the addition of glucanase, insoluble dextran formed by *S. mutans* was significantly decreased (Table 1). Amounts of insoluble glucan formed by *S. mutans* culture without the addition of glucanase and with the addition of glucanase were 0.6 and 0.24 μg/ml (61% reduction), respectively. Under co-cultivation of *S. mutans* and *E. faecium* at ratio of 3:1 (v/v), amounts of insoluble glucans formed without the addition of glucanase and with the addition of glucanase were, respectively, 0.3 and 0.2 μg/ml (32% reduction). Under co-cultivation of *S. mutans* and *E. faecium* at ratio of 1:1 (v/v), amounts of insoluble glucan formed without the addition of glucanase and with the addition of glucanase were 0.25 and 0.03 μg/ml (89% reduction), respectively. Ryu et al. (2000) reported that glucanase from *L. starkeyi* alone can hydrolyze insoluble-glucan of *S. mutans.* Stability of glucanase activity in the presence of *E. faecium* T7 is shown in Supplementary Table 3. During 14 days of cultutivation at room temperature (25 °C), *E. faecium* T7 amounts and glucanase activity were maintained at 10^9^ CFU/g and 22 U/g dextranase activity, respectively.

**Table 1:** Effect of adding glucanase on insoluble glucan formation in *S. mutans*/*E. faecium* T7 co-culture

Insoluble glucan (μg/ml)	*S. mutans*/*E. faecium* T7 ratio (v/v) co-culture			
	10:0	3:1	1:1	1:3
Without glucanase	0.6 ± 0.05^a^	0.3 ± 0.04^b^	0.25 ± 0.03^b^	0
With glucanase	0.24 ± 0.01^b^	0.21 ± 0.03^b^	0.03 ± 0.02^c^	0
Reduction %	61	32	89	–

Mean ± Standard error of the mean (SEM)

^a,b,c^Different superscripts in lower-case letter after values indicate significant difference at *p* < 0.05

### Inhibitory effect of *E. faecium* T7 on volatile sulfide compound production in *F. nucleatum* co-culture

**Fig. 3:**
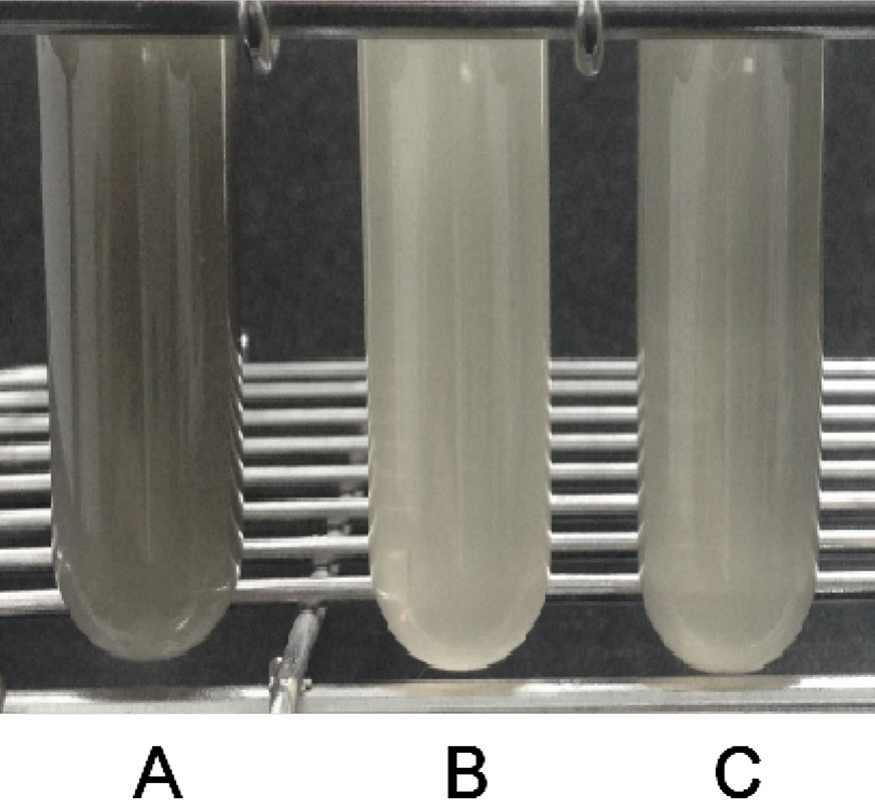
Effects of *E. faecium* T7 co-cultivation on the formation of hydrogen sulfide by *F. nucleatum*. (A) *F. nucleatum* single culture, (B) *E. faecium* single culture, (C) *F. nucleatum*/*E. faecium* co-culture. Black pigmentation is a marker for H_2_S production

Halitosis is a common problem in companion animals and humans. It is the first clinical sign of periodontal disease (Culham and Rawlings 1998). Many commercial oral rinses contain anti-plaque and anti-calculus ingredients such as chlorohexidine, cetylpyridinium chloride, alcohol, and/or zinc salts to prevent plaque and halitosis. The most effective and commonly used chemical is chlorhexidine. However, long-term use of chlorhexidine has side effects such as abnormal taste sensation, mucosal irritation, and tooth staining (Robinson 1995). In this study, the effect of *E. faecium* T7 on the reduction of VSCs by *F. nucleatum* in co-culture was determined by measuring iron sulfide (FeS) formation. *E. faecium* T7 inhibited the production of H_2_S by *F. nucleatum* in the co-culture, resulting in no black pigment formation in the culture supernatant. However, black pigments were observed in the single culture of *F. nucleatum* (Fig. 3). Thus, co-cultivation with *E. faecium* T7 can reduce the production of VSCs by *F. nucleatum*. It can also reduce the formation of insoluble glucan in sucrose medium produced by *S. mutans*. Therefore, *E. faecium* T7 co-culture can be used as a potentially effective method to improve canine oral health. Its effect can be expanded further by adding glucanase.

## Conclusion

The inhibitory effect of co-cultivation with *E. faecium* T7 in the presence of *L. starkeyi* glucanase (containing dextranase and mutanase equivalent activities) on insoluble glucan formation by *S. mutans* has been characterized for the first time. Co-cultivation of *F. nucleatum* with *E. faecium* T7 also decreased volatile sulfur compound produced by *F. nucleatum*. Therefore, *E. faecium* and glucanase can be used as potentially active ingredients of oral care products for pets by reducing plaque-forming bacteria growth and their by-products that cause cavity and periodontal disease.

## Electronic supplementary material

Below is the link to the electronic supplementary material.

Supplementary material 1 (DOCX 681 kb)
