# Supplementary material for: Decrease of insoluble glucan formation in Streptococcus mutans by co-cultivation with Enterococcus faecium T7 and glucanase addition
Source: Biotechnol Lett. 2017 Nov 21;40(2):375–81. doi: 10.1007/s10529-017-2478-z (PMC5813081; doi:10.1007/s10529-017-2478-z)
Supplement: Supplementary file 1 — Supplementary material 1 (DOCX 681 kb) [file 10529_2017_2478_MOESM1_ESM.docx]

**Supplementary material**

**Reduction of *Streptococcus mutans* insoluble glucan formation with *Enterococcus faecium* T7 co-cultivation and additional glucanase**

**Supplementary materials legends**

**Supplementary Table 1.** Biochemical characterization of the T7 isolate for the usage of 71 carbon sources

**Supplementary Table 2.** Biochemical characterization of the T7 isolate for the usage of 23 kinds of chemical sensitivity

**Supplementary Table 3.** The stability of *E. faecium* T7 and *L. starkeyi* mixture at room temperature

**Supplementary Fig. 1** The difference of colonial morphology between *S. mutans* (yellow arrow) and *E. faecium* (all colonies except yellow arrow) on BHI agar plate containing 50 g sucrose/l.

**Supplementary Table 1.** Biochemical characterization of the T7 isolate for the usage of 71 carbon sources

| No. | Carbon substrate | T7 | No. | Carbon substrate | T7 |
| --- | --- | --- | --- | --- | --- |
| 0 | Negative control | - | 36 | Gelatin | - |
| 1 | Dextrin | + | 37 | Glycyl-L-Prolin | - |
| 2 | D-Maltose | + | 38 | L-Alanine | - |
| 3 | D-Trehalose | + | 39 | L-Arginine | - |
| 4 | D-Cellobiose | + | 40 | L-Aspartic | - |
| 5 | Gentibiose | + | 41 | L-Glutamic Acid | - |
| 6 | Sucrose | + | 42 | L-Histidine | - |
| 7 | D-Turanose | - | 43 | L-Pyroglutamic Acid | - |
| 8 | Stachose | - | 44 | L-Serine | - |
| 9 | D-Raffinose | - | 45 | Pectin | B |
| 10 | α-D-Lactose | + | 46 | D-Galacturonic Acid | - |
| 11 | D-Melibiose | + | 47 | L-Galactonic Acid Lactone | - |
| 12 | β-Methyl-D-Glucoside | + | 48 | D-Gluconic Acid | + |
| 13 | D-Salicin | + | 49 | D-Glucuronic acid | B |
| 14 | N-Acetyl-D-Glucosamine | + | 50 | Glucuronamide | B |
| 15 | N-Acetyl-β-D-Mannosamine | B | 51 | Mucic Acid | - |
| 16 | N-Acetyl-D-Galactosamine | + | 52 | Quinic Acid | - |
| 17 | N-Acetyl Neuraminic Acid | - | 53 | D-Saccharic Acid | - |
| 18 | α-D-Glucose | + | 54 | p-Hydroxy-Phenylacetic Acid | - |
| 19 | D-Mannose | + | 55 | Methyl Pyruvate | - |
| 20 | D-Fructose | + | 56 | D-Lactic Acid Methyl Ester | - |
| 21 | D-Galactose | + | 57 | L-Lactic Acid | - |
| 22 | 3-Methyl Glucose | B | 58 | Citric Acid | - |
| 23 | D-Fucose | B | 59 | α-Keto-Glutaric Acid | - |
| 24 | L-Rhamnose | - | 60 | D-Malic Acid | - |
| 25 | L-Rhamnose | B | 61 | L-Malic Acid | - |
| 26 | Inosine | B | 62 | Bromo-Succinic Acid | - |
| 27 | D-Sorbitol | - | 63 | Tween 40 | - |
| 28 | D-Mannitol | + | 64 | γ-Amino-Butyric Acid | - |
| 29 | D-Arabitol | - | 65 | α-Hydroxy Butyric Acid | - |
| 30 | Myo-Inositol | - | 66 | β-Hydroxy-D,L Butyric Acid | - |
| 31 | Glycerol | - | 67 | α-Keto-Butyric Acid | B |
| 32 | D-Glucose-6-PO_4_ | - | 68 | Acetoaccetic Acid | B |
| 33 | D-Fructose-6-PO_4_ | B | 69 | Propionic Acid | - |
| 34 | D-Aspartic Acid | - | 70 | Acetic Acid | - |
| No. | Carbon substrate | T7 | No. | Carbon substrate | T7 |

Note: -, negative; +, positive; B, borderline. Biolog GEN III test results prepared based on usage of 71 carbon sources

**Supplementary Table 2.** Biochemical characterization of the T7 isolate for the usage of 23 kinds of chemical sensitivity

| No. | Carbon substrate | T7 | No. | Carbon substrate | T7 |
| --- | --- | --- | --- | --- | --- |
| 1 | Positive control | + | 13 | Lincomycin | - |
| 2 | pH 6 | + | 14 | Guanidine HCl | + |
| 3 | pH 5 | B | 15 | Niaproof 4 | - |
| 4 | 1% NaCl | + | 16 | Vancomycin | - |
| 5 | 4% NaCl | B | 17 | Tetrazolium Violet | + |
| 6 | 8% NaCl | B | 18 | Tetrazolium Blue | B |
| 7 | 1% Sodium Lactate | + | 19 | Nalidixic Acid | + |
| 8 | Fusidic Acid | - | 20 | Lithium Chloride | - |
| 9 | D-Serine | - | 21 | Potassium Tellurite | + |
| 10 | Troleandomycin | - | 22 | Aztreonam | + |
| 11 | Rifamycin SV | + | 23 | Sodium Butyrate | + |
| 12 | Minocycline | - | 24 | Sodium Bromate | B |

Note: -, negative; +, positive; B, borderline. Biolog GEN III test results prepared based on sensitivities with 23 chemicals

**Supplementary Table 3.** The stability of *E. faecium* T7 and *L. starkeyi* mixture at room temperature

|  | Time (day) | | |
| --- | --- | --- | --- |
| Stability | 0 | 7 | 14 |
| Log CFU/ml | 9.41 ± 0.1 | 9.34 ± 0.02 | 9.37 ± 0.04 |
| Dextranase equivalent Unit/ml | 0.42 | 0.42 | 0.42 |


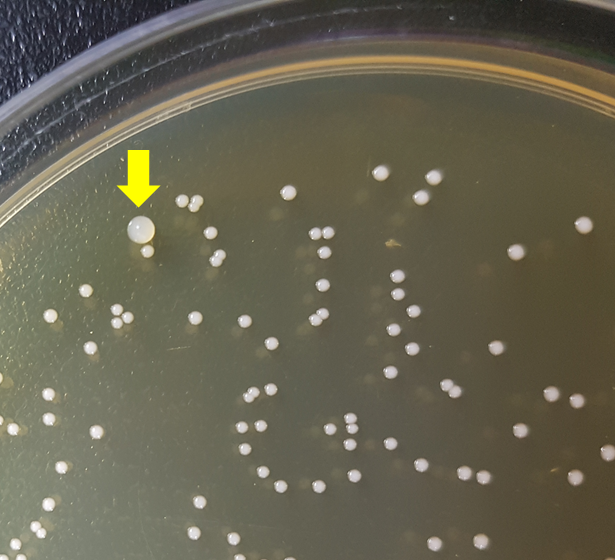


**Supplementary Fig. 1**
